# International Perspectives on Digital and Generative AI Adoption and Governance in Undergraduate Dental Education: A Cross-Sectional Survey

**DOI:** 10.3390/dj14020128

**Published:** 2026-02-23

**Authors:** Isabel C. Olegário, Niamh Coffey, Akhilanand Chaurasia, Albert Leung

**Affiliations:** 1 School of Dentistry, RCSI University of Medicine and Health Sciences, D02 YN77 Dublin, Ireland; niamhecoffey@rcsi.ie (N.C.); albertleung@rcsi.ie (A.L.); 2 Department of Oral Medicine and Radiology, King George’s Medical University, Lucknow 226003, India; akhilanandchaurasia@kgmcindia.edu

**Keywords:** dental education, generative artificial intelligence, AI governance, learning management systems, electronic dental records, patient management systems, assessment integrity

## Abstract

**Background/Objectives:** Digital technologies and generative artificial intelligence (AI) are increasingly used in undergraduate dental education, yet international variations in adoption and governance remain insufficiently described. This study aimed to characterise cross-national patterns of educational software use, perceived importance for curriculum delivery, and institutional readiness for AI governance. **Methods:** A cross-sectional online survey of educators and academic administrators involved in undergraduate dental education captured institutional software use across teaching delivery, learning management, assessment, clinical record systems, imaging, simulation, digital workflows, and generative AI. **Results:** A total of 97 respondents from 38 countries completed the survey, with most institutions delivering both undergraduate and postgraduate dental education (66.0%). Videoconferencing platforms were widely adopted. LMS provision varied, with Google Classroom, Moodle, and Blackboard most frequently reported. Paper-based clinical records remained in use in 32% of institutions. Among digital PMS/EDR platforms, axiUm, Salud/Titanium, and Carestream Dental were the most prevalent. Adoption of simulation software, CAD/CAM systems, and 3D printing was inconsistent. LMS and videoconferencing were most often rated as essential, whereas simulation, scanners, CAD/CAM, and 3D printing were generally considered useful but not essential. Generative AI use was commonly reported, while formal institutional guidance and policies were frequently absent. **Conclusions:** Although digital integration in undergraduate dental education is widespread, its distribution is uneven across different regions and technology domains. The combination of rapid generative AI uptake and limited governance highlights an urgent need for institution-level guidance, staff development, and strategic investment to support responsible and equitable integration.

## 1. Introduction

Digital technologies have become increasingly integral to the delivery of health professions education, reshaping the way curricula are designed, delivered, and evaluated [1]. In dentistry, software tools are now central to a wide range of educational processes, including online teaching and learning, electronic assessment, patient management, and simulation of clinical practice [2,3,4]. Learning management systems facilitate the organisation of curricula and provide platforms for blended education, videoconferencing supports synchronous and remote engagement, and electronic dental records familiarise students with digital workflows that they will encounter in practice. Advances in virtual reality simulation [5,6], and three-dimensional technologies such as CAD/CAM [7,8] and 3D printing [9,10] are expanding opportunities for preclinical and clinical training, whilst intraoral scanners and imaging platforms reflect the digital transformation of diagnostic and treatment modalities in modern dentistry.

Despite the growing importance of digital integration, there remains considerable variability in how software is adopted and implemented in undergraduate dental curricula worldwide [1,7,11]. Factors influencing uptake include institutional resources, faculty training, infrastructure, cost, and local educational priorities. In many low- or middle-income countries, barriers such as financial limitations and technological constraints impede widespread adoption, while even in resource-rich settings, challenges relating to interoperability, standardisation, and faculty readiness persist. This heterogeneity raises concerns regarding equity of educational experiences and preparedness of graduates to enter digitally driven clinical environments.

The emergence of generative artificial intelligence has added a new dimension to these debates. Tools such as ChatGPT, Microsoft Copilot, and Google Gemini are increasingly accessible to students and educators, offering novel opportunities for academic writing support, personalised learning, and teaching material development [12]. At the same time, their use raises questions regarding academic integrity, ethical standards, and the development of institutional policies to safeguard responsible application. Understanding how such technologies are being incorporated into undergraduate dental education globally is therefore essential for developing appropriate guidelines and ensuring alignment with educational objectives.

Existing literature on software integration in dental education is dominated by single-institution reports, region-specific studies, or evaluations of specific tools, with limited cross-national evidence spanning multiple software domains and institutional governance practices. As a result, there is insufficient empirical insight into which educational software domains are most commonly adopted across responding institutions, how institutions perceive the relative importance of these domains for curriculum delivery, and how institutions are responding to the rapid emergence of generative AI in the absence of consistent policy frameworks.

The present study objectives were to (i) describe the range of software domains used in undergraduate dental education internationally, (ii) summarise institutional perceptions of the importance of these domains for curriculum delivery, and (iii) describe reported generative AI adoption and the presence of institutional governance (policies or formal guidance).

The theoretical contribution of this work is to advance an understanding of digital readiness and governance gaps by mapping AI adoption across multiple educational and clinical software domains within a cross-national sample. The practical contribution is to provide a structured descriptive evidence base that can inform local institutional reflection and prioritisation in areas such as digital infrastructure, staff development, and policy development for responsible generative AI use.

## 2. Materials and Methods

### 2.1. Study Design and Setting

This investigation employed a cross-sectional survey design to characterise the integration of software tools in undergraduate dental education worldwide. Data were collected online using Microsoft Forms^®^ (Microsoft Corporation, Redmond, WA, USA) between 29 April 2025 and 21 July 2025. The study was designed and reported in accordance with the Consensus-Based Checklist for Reporting of Survey Studies (CROSS) guidelines [13].

### 2.2. Ethical Considerations

The study protocol was reviewed and approved by the Royal College of Surgeons in Ireland institutional Research Ethics Committee (Reference: REC202501013; date of approval: 28 March 2025) prior to data collection and analysis. At the survey entry page, participants were provided with a participant information package that included the study purpose, voluntariness of participation, and assurance of anonymity. Informed consent was obtained electronically through mandatory confirmation items before access to the questionnaire was granted.

### 2.3. Target Population, Eligibility Criteria, and Sampling

The target population comprised educators, clinical supervisors, and academic administrators involved in undergraduate dental education. Eligibility required current affiliation with a dental education provider and familiarity with institutional software used for teaching, assessment, and patient management.

Participation was voluntary and limited to one completed questionnaire per respondent in the final analytic dataset. A pragmatic, convenience sampling approach was used. Invitations were disseminated through institutional mailing lists, professional dental education networks, and targeted social media announcements to maximise geographic and institutional diversity. Respondents were targeted because they were more likely to have oversight of their respective institutional systems, procurement decisions, implementation processes, and governance (including policies relating to generative AI). In some institutions, the survey link may have been forwarded internally to the most appropriate respondent or completed following consultation with relevant colleagues to ensure accurate institutional reporting. No a priori sample-size calculation was performed because the study focused on descriptive mapping of global practices.

### 2.4. Questionnaire Development and Content

The survey instrument was developed by the research team to capture demographic and institutional characteristics and to map software usage across key domains of undergraduate dental education. The instrument comprised sections on institutional profile (institution, country, and education level) and software domains including videoconferencing platforms, learning management systems, patient management or electronic dental record systems, student daily clinical grading tools, student assessment platforms, dental imaging/radiographic software, simulation and virtual reality applications, intraoral scanner software, CAD/CAM systems, and three-dimensional printing.

Respondents rated the perceived essentiality of each software domain for undergraduate education using ordered categorical response options. These ratings were collected as institutional perceptions of relative importance for curriculum delivery and were not intended as validated measures of educational effectiveness or curricular necessity.

Additional items assessed generative artificial intelligence (AI) adoption, permitted user groups, stated purposes of use, and the presence of institutional policies or guidelines. The questionnaire concluded with an optional free-text item for comments and best practices. Response formats included single-choice, multiple-choice (multi-select), Likert-type ordinal ratings, and open responses. The instrument underwent internal review by the research team for face validity and clarity. No formal external pilot test was conducted. The complete questionnaire is available at: https://forms.office.com/e/BSUPrSHntf, accessed on 11 February 2026.

### 2.5. Survey Administration and Procedures

The survey was administered via a public link hosted in Microsoft Forms. Potential participants received information about the study aims, voluntary participation, anonymity, data protection, and investigator contact details on the landing page. Respondents first completed three consent confirmations indicating that they had read and understood the information provided, agreed to the anonymised use of their responses for research, and understood they could withdraw at any point before final submission. The survey allowed anonymous participation; Microsoft Forms^®^ did not collect names or institutional emails by default, and IP addresses were not stored in the analytic dataset. To mitigate multiple submissions, we performed post hoc screening for duplicate entries based on the institution/country data and retained only a single record when entries were flagged during data cleaning. The median questionnaire completion time was 5.7 min (interquartile range 5.1 min), based on the difference between recorded start and completion timestamps.

### 2.6. Outcomes and Variables

Primary outcomes comprised the reported use and perceived essentiality of software across predefined educational domains. Secondary outcomes included reported advantages and limitations of patient management and electronic dental record systems, adoption of simulation technologies, CAD/CAM systems, and three-dimensional printing, as well as institutional practices and governance related to generative artificial intelligence. Institutional context was characterised by country and level of provision (undergraduate, postgraduate, or both). Multi-select items captured platform brands and system names, while Likert-type ordinal responses were used to assess perceived essentiality. Open-ended items provided qualitative insights into implementation experiences and examples of best practice. Importantly, “generative AI use” was captured as a respondents-reported institutional presence of use (for example, use by staff and or students for education-related activities) rather than as a quantified measure of frequency or intensity. The survey did not assess how often AI tools were used, the proportion of users within institutions, or whether use was formally supported through institutional policy versus informal or ad hoc practice.

### 2.7. Handling of Duplicate Entries, Missing Data, and Non-Response

Entries flagged as duplicates during data cleaning were removed prior to analysis; the final analytic sample comprised 97 unique submissions from 38 countries. Missingness in the optional free-text item was common, whereas core categorical items (including essentiality ratings) were largely complete. Because missingness in key variables was minimal, complete-case analysis was applied for descriptive summaries on a per-item basis. The survey distribution strategy precluded a reliable denominator of individuals reached; as such, view, participation, and completion proportions and a conventional response rate could not be estimated. No post-stratification weights were applied.

### 2.8. Statistical Analysis

Data were exported to comma-separated values and analysed descriptively. Statistical analyses were performed in Stata, Release 18 (StataCorp LLC, College Station, TX, USA) and figures were prepared from the aggregated outputs. Categorical variables were summarised as frequencies and percentages. Ordinal essentiality ratings were summarised by category distributions within each software domain.

## 3. Results

**Figure 1:**
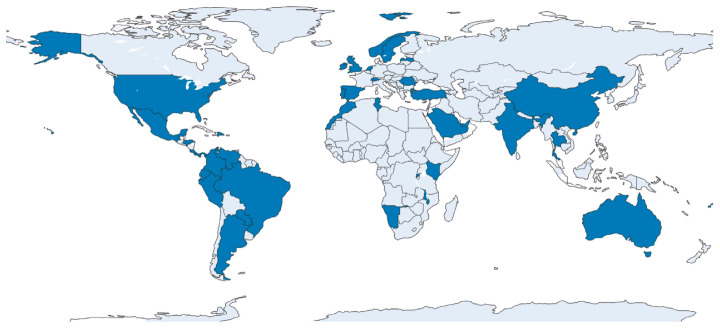
Geographic distribution of the 96 dental schools from 38 countries that responded to the survey.

A total of 97 respondents in 38 countries completed the survey (Figure 1). The largest number of responses came from India (*n* = 32, 33.0%), followed by Mexico (*n* = 4, 4.1%), Brazil (*n* = 4, 4.1%), and Australia (*n* = 3, 3.1%). Most participating institutions offered both undergraduate and postgraduate education (*n* = 64, 66.0%), while 20 institutions (20.6%) provided undergraduate programmes only and 13 (13.4%) offered postgraduate training exclusively.

Videoconferencing platforms were widely used across institutions. Zoom was the most frequently reported (*n* = 20, 20.6%), often in combination with Google Meet (*n* = 13, 13.4%) or Microsoft Teams (*n* = 6, 6.2%). Google Meet and Microsoft Teams were also commonly used independently or in combination. Learning management systems (LMS) varied considerably. Google Classroom was most frequently cited (*n* = 12, 12.4%), followed by Moodle (*n* = 10, 10.3%) and Blackboard (*n* = 8, 8.2%). Seven respondents reported having no LMS in place.

A total of 22 different Patient Management Systems (PMS) and Electronic Dental Record (EDR) platforms were reported across participating dental schools. The most frequently used systems were axiUm (*n* = 12), Salud (*n* = 11), Carestream Dental (*n* = 10), SMILE (*n* = 6), and Titanium (*n* = 4). Less commonly reported systems included Dentrix, EXACT, HIS, ERP, Dentium Edu, ROMEU (Internal), and others, each mentioned by one to three institutions. Table 1 illustrates the advantages and limitations listed for each PMS. Across systems, the most consistently cited advantages were student clinic organisation, real-time updates, integration with radiographic software, enhanced patient data security, streamlined treatment planning, billing and administrative support, and control of student/supervisor activities. In contrast, common limitations included technical issues, steep learning curves, high costs of software or maintenance, poor or limited technical support, slow system performance, system crashes or downtime, and limited functionality. Some region-specific challenges, such as low acceptance among older clinicians and cybersecurity concerns, were also highlighted.

A wide variation in systems for student daily clinical grading was observed across participating institutions. The majority still relied on paper-based grading (*n* = 50). Among digital solutions, CAFS (*n* = 11), Salud (*n* = 10), and AxiUm (*n* = 9) were the most commonly used. Less frequently reported systems included OSLER (*n* = 4), REXX (*n* = 2), and single instances of Google Forms, Sis, Eportfolio, UAG, CAFS and Salud, SMILE, and other non-specified approaches.

A variety of online assessment platforms were reported across institutions. The most widely used was Moodle (*n* = 21), followed by Blackboard (*n* = 14) and ExamSoft (*n* = 10). Other platforms with multiple mentions included TestReach (*n* = 5), Risr/assess (*n* = 5), Synap (*n* = 4), and Inspera (*n* = 4). Less frequently reported systems were Canvas, SMILE, Ans, TestVision, Questionmark, Socrative, Zawin, Scorion, REXX, ExamPlus, Turnitin, and Respondus Lockdown Browser, each cited by a single institution.

A wide range of radiographic software platforms were reported. The most frequently used were Carestream (*n* = 22), Romexis (*n* = 19), DEXIS (*n* = 13), and Sidexis (*n* = 12). Other systems were cited less commonly, including MiPACS (*n* = 3), SOTA (*n* = 2), and Dolphin/Nemotec (*n* = 2). Single-institution reports included EDent-i, Digora/Demexis, Dimaxis, Belmont, Vista Scan, VistaSoft, Examine Pro, Infinitt, Digora, DENTIS, Emago, and DBSWIN.

The use of simulation and virtual reality software in undergraduate teaching was inconsistent. Almost two-thirds of respondents reported no use of simulation systems (*n* = 63, 64.9%). Among those that did, Simodont was most frequently employed (*n* = 11, 11.3%), followed by Dentsim and PerioSim. Among participating institutions, the most widely reported intraoral scanner systems were 3Shape TRIOS (*n* = 28), CEREC (*n* = 25), and iTero (*n* = 23). Other systems were cited less frequently, including Planmeca Emerald/PlanScan (*n* = 9), Medit (*n* = 7), Carestream *(n* = 5), and Straumann (*n* = 2). A further 33 institutions (33%) reported not using intraoral scanners.

In relation to CAD/CAM technologies, 45 institutions (46.4%) reported no access. Where present, the most commonly used systems were CEREC (*n* = 36), 3Shape (*n* = 15), Planmeca PlanCAD (*n* = 10), and Exocad (*n* = 9). Three-dimensional printing was also reported to be infrequently adopted, with 54 institutions (55.7%) reporting no access. Among those that had implemented such systems, Formlabs (*n* = 7, 7.2%) and Asiga (*n* = 4, 4.1%) were the most commonly used brands.

Respondents rated the perceived importance of different categories of software in undergraduate dental education (Table 2). Learning management systems and videoconferencing platforms were most frequently considered essential, followed by patient management systems and dental imaging software. In contrast, simulation, intraoral scanning, CAD/CAM systems, and three-dimensional printing were more often rated as useful but not essential.

**Figure 2:**
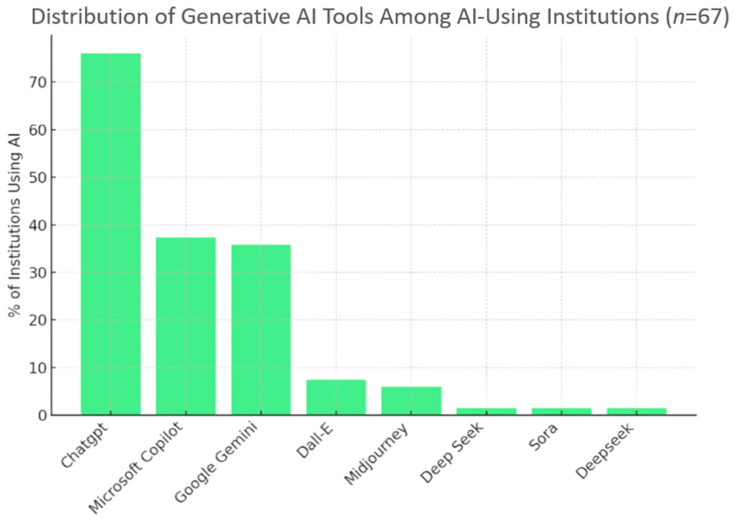
Distribution of Generative AI Tools among AI-using institutions (*n* = 67).

Generative artificial intelligence was reported as in use by 71 institutions (73.2%). ChatGPT was the most frequently used tool (*n* = 11, 11.3%), often in combination with Google Gemini or Microsoft Copilot (Figure 2).

**Figure 3:**
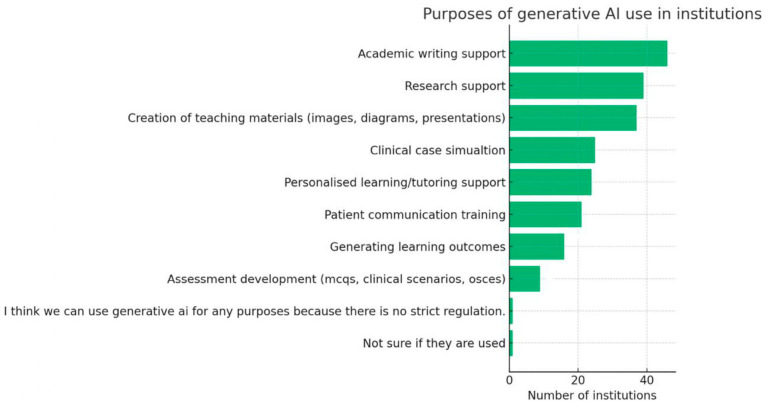
Purposes of generative AI use in institutions.

Twenty-six respondents (26.8%) reported no current use of generative AI. The most common uses of generative AI in institutions are academic writing support (46 institutions), research support (39), and creation of teaching materials (37) (Figure 3). Most institutions permitted use by both staff and students, although 65 respondents (67.0%) indicated that no formal institutional policy or guidelines existed regarding generative AI.

As summarised in Table 3, these findings provide direct signals for institutional decision-making across procurement, governance, curriculum design, and assessment, while also identifying areas that warrant further empirical investigation.

## 4. Discussion

This international survey provides a broad snapshot of software integration in undergraduate dental education across 38 countries and 97 institutions. The sample size and geographic spread strengthen the descriptive value of the findings and enable comparisons across major software domains used in teaching, assessment, and patient care. Nonetheless, participation patterns were uneven, with responses clustering in South Asia and Latin America, and limited representation from sub-Saharan Africa, parts of Eastern and Central Europe, and Oceania. While a large, diverse sample enhances the credibility of cross-sectional descriptions, gaps in geographic coverage limit inferences about global uniformity and might mask regional heterogeneity in access, infrastructure, and policy environments. As a convenience sample, participation was expected to be uneven across regions, and does not support claims of global representativeness. Findings have therefore been contextualised and interpreted as regionally influenced patterns observed within an international paradigm.

Videoconferencing platforms and learning management systems (LMS) emerged as the most entrenched technologies in undergraduate dental education. This finding aligns with the sector’s accelerated adoption of remote and blended delivery during and after the COVID-19 pandemic [14,15,16]. Continued reliance on Zoom, Microsoft Teams, and Google Meet suggests that synchronous online engagement has become embedded in routine pedagogical practice rather than serving only as an emergency measure. Similarly, LMS such as Moodle, Canvas, and Blackboard were consistently rated as “essential,” reflecting their central role in course orchestration, asynchronous learning, communication, and assessment workflows.

Electronic Dental Records (EDR) and Patient Management Systems (PMS) were widely reported but not yet universal, with many institutions still relying on paper-based records. Where implemented, digital systems were valued for case organisation, data security, and real-time access, while cost, training requirements, technical reliability, and interoperability remained significant barriers. The strategic potential of cloud-enabled EDR/PMS lies in secure role-based access and auditability, as well as integration with imaging, grading, and analytics to streamline clinical education and quality assurance. However, successful adoption depends on robust governance, vendor due diligence, and faculty development to avoid fragile deployments and uneven student experiences.

Adoption of simulation and extended reality was highly variable and, in many institutions, absent. Where available, simulators were recognised for supporting deliberate practice and structured feedback in preclinical training. Similar trends were observed for intraoral scanners, CAD/CAM systems, and three-dimensional printing: their use is increasing but far from universal. These technologies offer students exposure to contemporary restorative and prosthodontic workflows and foster collaboration with dental technology, yet barriers include high cost, maintenance, and limited curricular integration. Without protected time, staff upskilling, and technical support, these tools risk remaining peripheral add-ons rather than transformative components of education [5,6,17]. Institutions that lack resources may face widening disparities in preparing graduates for digitally enabled practice.

Perceptions of “essentiality” reflected a pragmatic hierarchy. Tools that directly enable course delivery and management (videoconferencing and LMS) were most often deemed essential, followed by those linked to patient care documentation and radiographic interpretation. By contrast, simulation platforms, intraoral scanners, CAD/CAM, and 3D printing were more often rated as “useful but not essential.” This prioritisation reflects cost profiles, infrastructure requirements, and the perception that core learning outcomes can be achieved through lower-tech alternatives.

Generative artificial intelligence (AI) was already in widespread use [18,19,20]. ChatGPT was most frequently cited, alongside tools such as Microsoft Copilot and Google Gemini. Reported applications ranged from drafting instructional materials and providing formative feedback to research support and administrative streamlining, demonstrating AI’s versatility across teaching, assessment design, and student support. However, most institutions lacked formal policies. This governance gap may be associated with inconsistent practices and may increase risks relating to assessment integrity, privacy and data protection, and inequities in access and support, as highlighted in the wider literature [12]. Permissions for AI use also varied between staff, students, and administrators, underscoring the need for harmonised approaches [20,21].

Emerging evaluation studies further demonstrate that large language models vary substantially in their performance when answering dental student assessment items, raising important concerns regarding assessment validity, fairness, and consistency if access and expectations are not clearly defined [22]. Discipline-specific educational research also highlights the expanding relevance of artificial intelligence across multiple areas of clinical teaching, including endodontics [23], restorative dentistry [24], prosthodontics [25], and implant dentistry [26]. Across these domains, proposed applications extend beyond radiographic interpretation to include case difficulty assessment, diagnostic reasoning support, treatment planning and sequencing, materials selection, simulation-based skill development, real-time clinical guidance, outcome prediction, and personalised formative feedback. Collectively, these developments underscore the need for robust governance frameworks and deliberate curriculum design to ensure that AI is integrated in ways that are safe, equitable, transparent, and educationally meaningful.

Responsible integration requires both policy and capacity building. Institutions must establish clear policies defining acceptable use, transparency in disclosure, data protection safeguards, and alignment with assessment integrity. At the same time, staff and students need targeted training to develop a critical understanding of AI’s limitations, biases, and responsible prompting. Mature digital ecosystems are well placed to leverage AI for personalised learning, clinical reasoning, and efficiency gains, while under-resourced contexts risk being further disadvantaged if AI replaces, rather than augments, high-quality teaching. Future directions should include staged rollouts, continuous evaluation of educational impact, and cross-institutional sharing of policies and frameworks to harmonise practices and reduce duplication of effort.

Decisions about whether, when, and how to integrate AI into dental education shape not only teaching practices but also graduates’ future clinical decision-making. Exposure to AI tools without appropriate governance risks normalising over-reliance, bias, and inconsistent documentation standards, while restrictive approaches may leave students unprepared for the digital environments they will encounter in practice. To safeguard future clinical care, curricula should explicitly embed three principles: human-in-the-loop, ensuring that students understand clinicians remain accountable for all final decisions; auditability, promoting the practice of saving and disclosing AI prompts/outputs in ways consistent with clinical logbooks and data protection standards; and calibration, encouraging structured comparison of AI outputs against guidelines and expert judgement to avoid both overtrust and neglect. Embedding these principles in education will better prepare graduates to use AI responsibly, critically, and ethically in patient care.

This study used convenience sampling and relied on self-reported institutional responses, which might be influenced by respective respondent roles, local interpretations of terminology, and recall. Participation was uneven across different regions, limiting external validity and precluding true global representativeness. Findings are therefore contextually interpreted as regionally influenced patterns observed within an international sample. The cross-sectional design does not support causal inference, and reported generative AI “use” was not quantified in terms of frequency, depth, or formal institutional endorsement. In addition, the perceived importance ratings reflect perceptions rather than validated measures of educational effectiveness. Future research should include longitudinal and comparative designs to evaluate how adoption evolves, what implementation strategies are effective, and how governance approaches influence educational outcomes. Mixed-methods studies incorporating policy review, structured interviews, objective indicators of usage, and training provision would strengthen understanding of how digital and AI tools are being integrated, regulated, and evaluated across a diverse context.

Within these constraints, the survey suggests that technologies supporting educational delivery and management are now foundational, while digital clinical workflows and simulation remain unevenly integrated. The rapid uptake of generative AI against a backdrop of limited policy infrastructure highlights an urgent need for governance, harmonised international guidance, and strategic investment [11,18,19]. A coordinated approach that combines cloud-ready records, interoperable imaging, simulation, and digital fabrication with robust AI policies and faculty development offers a realistic pathway to narrowing disparities and aligning undergraduate programmes with contemporary dental practice.

Finally, there is a pressing need for international consensus on the role of AI in dental education, particularly in student assessment, to safeguard fairness, integrity, and educational value. Future educational research should systematically evaluate the impact of AI on learning outcomes, assessment practices, and professional formation, moving the debate from adoption to demonstrable, evidence-based benefit.

## 5. Conclusions

This international survey demonstrates that digital software integration in undergraduate dental education is widespread but uneven, with foundational platforms such as learning management systems and videoconferencing now well established. In contrast, digital clinical workflows, simulation technologies, and advanced manufacturing tools remain variably adopted and often peripheral to curricula. The rapid uptake of generative artificial intelligence in the absence of formal institutional policies highlights a critical governance gap. Strategic investment, faculty development, and harmonised international guidance are required to support equitable, ethical, and effective digital and AI integration in dental training.

## Figures and Tables

**Table 1:** Frequency, Reported Advantages, and Limitations of Patient Management and Electronic Dental Record Systems Across Dental Schools.

PMS/EDR System	Frequency (*n*)	Core Reported Advantages (Thematic)	Common Reported Limitations (Thematic)
axiUm	12	Clinic organisation and supervision control; radiographic integration; real-time updates; data security	High cost; steep learning curve; technical instability; limited customisation
Salud	11	Clinic organisation; radiographic integration; data security; real-time updates	High cost; limited functionality; technical instability; learning curve
Carestream Dental	10	Clinic organisation; radiographic integration; treatment planning support; data security	High cost; technical instability; limited functionality; support issues
SMILE	6	Remote record access; clinic organisation; radiographic integration; supervision control	High cost; technical instability; learning curve; variable clinician acceptance
Titanium	4	Clinic organisation; radiographic integration; supervision control	Limited functionality; poor usability for dental charting; technical instability
Dentrix	3	Radiographic integration; data security; clinic organisation	High cost; technical issues
ERP	2	Administrative support; clinic organisation; real-time updates	Outdated design; data loss risk; limited functionality
EXACT	2	Treatment planning; data security	Limited supervision control; technical instability; support issues
HIS	2	Administrative and clinical integration; data analytics; radiographic integration	High cost; technical instability; support issues
ROMEU (Internal)	1	Supervision control; radiographic integration; data security	Limited functionality; technical instability; learning curve
Dentium Edu	1	Clinic organisation; radiographic integration	Limited functionality; performance issues
Newsoft DS	1	Clinic organisation; radiographic integration	Technical instability; learning curve
SKaPa	1	Supervision control; data security; radiographic integration	High cost; technical issues
Trtek	1	Administrative support; real-time updates	Limited functionality; technical instability
SmartMedical	1	Data security; real-time updates	Technical issues
Dentally	1	Treatment planning	Learning curve
SIS	1	Supervision control; user-friendly interface	Limited functionality; support issues
Dentalink	1	Radiographic integration	High cost
Dentidesk	1	Clinic organisation; supervision control; radiographic integration	High cost
Zawin	1	Clinic organisation; radiographic integration; data security	Cybersecurity concerns; learning curve
Opus Dental	1	Clinic organisation; data security	Limited functionality; performance issues
MEDERP	1	Clinic organisation; radiographic integration; supervision control	Learning curve

**Table 2:** Perceived importance of different types of software for undergraduate dental education (N = 97).

Software Type	Essential *n* (%)	Useful But Not Essential *n* (%)	Not Essential *n* (%)
Videoconferencing platforms	69 (71.1%)	25 (25.8%)	3 (3.1%)
Learning Management System (LMS)	78 (80.4%)	17 (17.5%)	2 (2.1%)
Patient Management System (PMS)	80 (82.5%)	15 (15.5%)	2 (2.1%)
Student Daily Clinical Grading System	71 (73.2%)	23 (23.7%)	3 (3.1%)
Dental imaging/ Radiographic software tools	86 (88.7%)	10 (10.3%)	1 (1.0%)
Dental simulation software/ Virtual reality simulators	55 (56.7%)	38 (39.2%)	4 (4.1%)
Intraoral scanner software	76 (78.4%)	19 (19.6%)	2 (2.1%)
Assessment software/ Online examination platforms	66 (68.0%)	26 (26.8%)	5 (5.2%)
CAD/CAM systems	70 (72.2%)	24 (24.7%)	3 (3.1%)
3D printing technologies	62 (63.9%)	30 (30.9%)	5 (5.2%)

**Table 3:** Mapping Findings to Decisions.

Key Finding	Decision It Informs
High AI use alongside lack of institutional policies	Issue interim guidance; redesign exam rules (AI disclosure); prioritise staff/student AI training
Videoconferencing and LMS consistently rated as “essential”	Maintain licencing as core infrastructure; embed in curriculum delivery and contingency planning
Many schools still using paper-based records	Prioritise procurement of PMS/EDR with audit trails; allocate training and change-management resources
Radiographic software widely used and seen as essential	Budget for licences and integration with PMS/EDR; ensure interoperability with imaging and grading systems
Simulation, scanners, CAD/CAM, and 3D printing adoption highly variable	Phase investments aligned with learning outcomes; consider shared facilities or industry partnerships; develop future studies to compare effectiveness of simulation versus traditional training tools to inform cost–benefit decisions.
Student daily clinical grading often paper-based	Digitise supervision/feedback systems; align platforms with assessment analytics and competency tracking
Permissions for AI use vary across staff and students	Harmonise governance; define permitted use cases; implement data protection safeguards

## Data Availability

The original contributions presented in this study are included in the article. Further inquiries can be directed to the corresponding author.

## Abbreviations

The following abbreviations are used in this manuscript:

**Abbreviation**	**Definition**
AI	Artificial intelligence
CAD/CAM	Computer-aided design/computer-aided manufacturing
EDR	Electronic dental record
LMS	Learning management system
PMS	Patient management system
VR	Virtual reality
